# Structure and function of human Naa60 (NatF), a Golgi-localized bi-functional acetyltransferase

**DOI:** 10.1038/srep31425

**Published:** 2016-08-23

**Authors:** Ji-Yun Chen, Liang Liu, Chun-Ling Cao, Mei-Jun Li, Kemin Tan, Xiaohan Yang, Cai-Hong Yun

**Affiliations:** 1 Department of Biophysics, School of Basic Medical Sciences, Peking University Health Science Center, Beijing 100191, P. R. China; 2 Institute of Systems Biomedicine and Beijing Key Laboratory of Tumor Systems Biology, School of Basic Medical Sciences, Peking University Health Science Center, Beijing 100191, P. R. China; 3 Structural Biology Center, Biosciences, Argonne National Laboratory, 9700 S. Cass Avenue, Argonne, IL 60439, USA; 4 Key Laboratory of Carcinogenesis and Translational Research (Ministry of Education), Department of Biochemistry and Molecular Biology, Peking University Health Science Center, Beijing 100191, China

## Abstract

N-terminal acetylation (Nt-acetylation), carried out by N-terminal acetyltransferases (NATs), is a conserved and primary modification of nascent peptide chains. Naa60 (also named NatF) is a recently identified NAT found only in multicellular eukaryotes. This protein was shown to locate on the Golgi apparatus and mainly catalyze the Nt-acetylation of transmembrane proteins, and it also harbors lysine N^ε^-acetyltransferase (KAT) activity to catalyze the acetylation of lysine ε-amine. Here, we report the crystal structures of human Naa60 (hNaa60) in complex with Acetyl-Coenzyme A (Ac-CoA) or Coenzyme A (CoA). The hNaa60 protein contains an amphipathic helix following its GNAT domain that may contribute to Golgi localization of hNaa60, and the β7-β8 hairpin adopted different conformations in the hNaa60(1-242) and hNaa60(1-199) crystal structures. Remarkably, we found that the side-chain of Phe 34 can influence the position of the coenzyme, indicating a new regulatory mechanism involving enzyme, co-factor and substrates interactions. Moreover, structural comparison and biochemical studies indicated that Tyr 97 and His 138 are key residues for catalytic reaction and that a non-conserved β3-β4 long loop participates in the regulation of hNaa60 activity.

Acetylation is one of the most ubiquitous modifications that plays a vital role in many biological processes, such as transcriptional regulation^1^, protein-protein interaction^2^, enzyme activity^3^, protein stability^4^, antibiotic resistance^5^, biological rhythm^6^ and so on. Protein acetylation can be grouped into lysine N^ε^-acetylation and peptide N-terminal acetylation (Nt-acetylation). Generally, N^ε^-acetylation refers to the transfer of an acetyl group from an acetyl coenzyme A (Ac-CoA) to the ε-amino group of lysine^7^. This kind of modification is catalyzed by lysine acetyltransferases (KATs), some of which are named histone acetyltransferases (HATs) because early studies focused mostly on the post-transcriptional acetylation of histones^8^.

Despite the prominent accomplishments in the field regarding N^ε^-acetylation by KATs for over 50 years, the significance of the more evolutionarily conserved Nt-acetylation is still inconclusive. Nt-acetylation is an abundant and evolutionarily conserved modification occurring in bacteria, archaea and eukaryotes^9^. It is estimated that about 80–90% of soluble human proteins and 50–70% of yeast proteins are subjected to Nt-acetylation, where an acetyl moiety is transferred from Ac-CoA to the α-amino group of the first residue^10^. Recently Nt-acetylome expands the Nt-acetylation to transmembrane proteins^11^. Unlike N^ε^-acetylation that can be eliminated by deacetylases, Nt-acetylation is considered irreversible since no corresponding deacetylase is found to date^12^. Although Nt-acetylation has been regarded as a co-translational modification traditionally, there is evidence that post-translational Nt-acetylation exists^9^,^13^. During the past decades, a large number of Nt-acetylome researches have shed light on the functional roles of Nt-acetylation, including protein degradation, subcellular localization, protein-protein interaction, protein-membrane interaction, plant development, stress-response and protein stability^14^,^15^.

The Nt-acetylation is carried out by N-terminal acetyltransferases (NATs) that belong to the GNAT superfamily. To date, six NATs (NatA/B/C/D/E/F) have been identified in eukaryotes. About 40 percent of Nt-acetylation of soluble proteins in cells is catalyzed by NatA complex which is composed of the catalytic subunit Naa10p and the auxiliary subunit Naa15p^9^. NatE was found to physically interact with the NatA complex without any observation of impact on NatA-activity^12^. Two other multimeric complexes of NATs are NatB and NatC which contain the catalytic subunits Naa20 and Naa30 and the auxiliary subunits Naa25 and Naa35/Naa38, respectively^14^. Furthermore, only the catalytic subunits Naa40 and Naa60 were found for NatD and NatF, respectively^9^. Besides Nt-acetylation, accumulating reports have proposed N^ε^-acetylation carried out by NATs^12^,^16^,^17^.

There is an evolutionary increasing in the degree of Nt-acetylation between yeast and human which could partly be explained by the contribution of NatF^18^. As the first N-terminal acetyltransferase discovered on an organelle, NatF, encoded by *NAA60* and also named as Histone acetyltransferase type B protein 4 (HAT4), Naa60 or N-acetyltransferase 15 (NAT15), is the youngest member of the NAT family^11^,^17^,^18^. Unlike other NATs that are highly conserved among lower and higher eukaryotes, NatF only exists in higher eukaryotes^12^,^18^. Subsequent researches indicated that NatF displays its catalytic ability with both Nt-acetylation and lysine N^ε^-acetylation^17^,^18^. As an N-terminal acetyltransferase, NatF can specifically catalyze acetylation of the N-terminal α-amine of most transmembrane proteins and has substrate preference towards proteins with Met-Lys-, Met-Val-, Met-Ala- and Met-Met-N-termini, thus partially overlapping substrate selectivity with NatC and NatE^18^. On the other hand, NatF, with its lysine acetyltransferase activity, mediates the lysine acetylation of free histone H4, including H4K20, H4K79 and H4K91^17^. Another important feature of NatF is that this protein is anchored on the Golgi apparatus through its C-terminal membrane-integrating region and takes part in the maintaining of Golgi integrity^11^,^17^. With its unique intracellular organellar localization and substrate selectivity, NatF appears to provide more evolutionary information among the NAT family members.

It was recently found that NatF facilitates nucleosomes assembly and that *NAA60* knockdown in MCF7-cell inhibits cell proliferation, sensitizes cells to DNA damage and induces cell apoptosis^17^. In *Drosophila* cells, *NAA60* knockdown induces chromosomal segregation defects during anaphase including lagging chromosomes and chromosomal bridges^18^. Much recent attention has also been focused on the requirement of NatF for regulation of organellar structure. In HeLa cells, *NAA60* knockdown causes Golgi apparatus fragmentation which can be rescued by overexpression Naa60^11^. The systematic investigation of publicly available microarray data showed that NATs share distinct tissue-specific expression patterns in *Drosophila* and NatF shows a higher expression level in central nervous system of *Drosophila*^19^.

In this study, we solved the structures of human Naa60 (NatF) in complex with coenzyme. The hNaa60 protein contains a unique amphipathic α-helix (α5) following its GNAT domain that might account for the Golgi localization of this protein. Crystal structures showed that the β7-β8 hairpin rotated about 50 degrees upon removing the C-terminal region of the protein and this movement substantially changed the geometry of the substrate-binding pocket. Remarkably, we find that Phe 34 may participate in the proper positioning of the coenzyme for the transfer reaction to occur. Further structure comparison and biochemical studies also identified other key structural elements essential for the enzyme activity of Naa60.

## Results

### Overall structure of hNaa60

**Figure 1:**
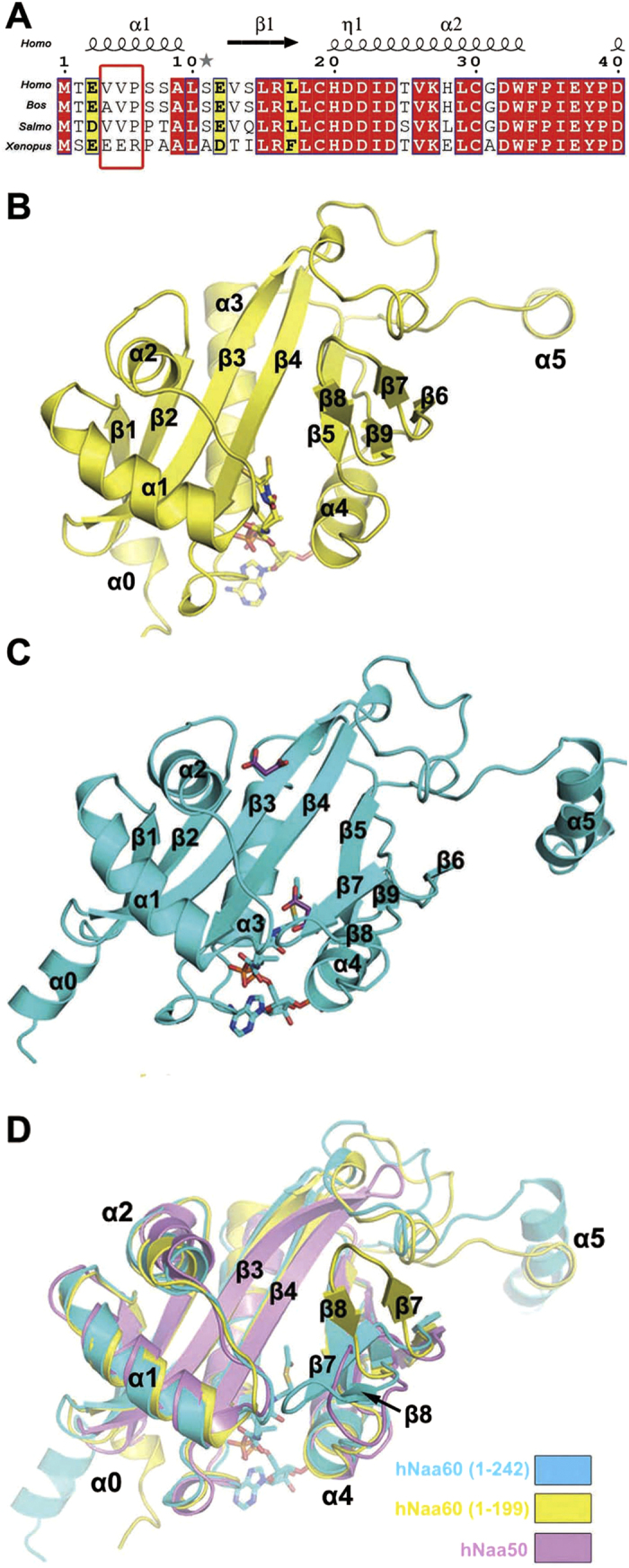
Overall structure of Naa60. (**A**) Sequence alignment of Naa60 (NatF, HAT4) from different species including *Homo sapiens (Homo*), *Bos mutus (Bos*), *Salmo salar (Salmo*) and *Xenopus (Silurana) tropicalis (Xenopus*). Alignment was generated using NPS@ and ESPript.3.0 (http://espript.ibcp.fr/ESPript/ESPript/). Residues 4–6 are highlighted in red box. (**B**) The structure of hNaa60(1-199)/CoA complex is shown as a yellow cartoon model. The CoA molecule is shown as sticks. (**C**) The structure of hNaa60(1-242)/Ac-CoA complex is presented as a cartoon model in cyan. The Ac-CoA and malonate molecules are shown as cyan and purple sticks, respectively. The secondary structures are labeled starting with α0. (**D**) Superposition of hNaa60(1-242) (cyan), hNaa60(1-199) (yellow) and hNaa50 (pink, PDB 3TFY). The Ac-CoA of hNaa60(1-242)/Ac-CoA complex is represented as cyan sticks.

In the effort to prepare the protein for structural studies, we tried a large number of hNaa60 constructs but all failed due to heavy precipitation or aggregation. Sequence alignment of Naa60 from different species revealed a Glu-Glu-Arg (EER) versus Val-Val-Pro (VVP) sequence difference near the N-terminus of the protein in *Xenopus Laevis* versus *Homo sapiens* (Fig. 1A). Considering that terminal residues may lack higher-order structure and hydrophobic residues in this region may expose to solvent and hence cause protein aggregation, we mutated residues 4–6 from VVP to EER for the purpose of improving solubility of this protein. According to previous studies, this N-terminal region should not interfere with hNaa60’s Golgi localization^11^. We tried many hNaa60 constructs with the three-residues mutation but only the truncated variant 1-199 and the full-length protein behaved well. We obtained the crystal of the truncated variant 1-199 in complex with CoA first, and after extensive trials we got the crystal of the full-length protein (spanning residues 1-242) in complex with Ac-CoA (Fig. 1B,C). Hereafter, all deletions or point mutants of hNaa60 we describe here are with the EER mutation. The crystal structures of hNaa60(1-242)/Ac-CoA and hNaa60(1-199)/CoA were determined by molecular replacement and refined to 1.38 Å and 1.60 Å resolution, respectively (Table 1). The electron density maps were of sufficient quality to trace residues 1-211 of hNaa60(1-242) and residues 5-199 of hNaa60(1-199).

The structure of hNaa60 protein contains a central domain exhibiting a classic GCN5-related N-acetyltransferase (GNAT) folding, along with the extended N- and C-terminal regions (Fig. 1B,C). The central domain includes nine β strands (β1-β9) and four α-helixes (α1-α4) and is highly similar to the known hNaa50p and other reported NATs (Fig. 1D). However, in hNaa60, there is an extra 20-residue loop between β3 and β4 that forms a small subdomain with well-defined 3D structure (Fig. 1B–D). Furthermore, the β7-β8 strands form an approximately antiparallel β-hairpin structure remarkably different from that in hNaa50p (Fig. 1D). The N- and C-terminal regions form helical structures (α0 and α5) stretching out from the central GCN5-domain (Fig. 1C).

Interestingly, we found that the catalytic activity of hNaa60(1-242) is much lower than that of hNaa60(1-199) (Figure S1), indicating that residues 200–242 may have some auto-inhibitory effect on the activity of the enzyme. However, since this region was not visible in the hNaa60(1-242) crystal structure, we do not yet understand how this happens. Another possibility is that since hNaa60 is localized on Golgi apparatus, the observed low activity of the full-length hNaa60 might be related to lack of Golgi localization of the enzyme in our *in vitro* studies. For the convenience of studying the kinetics of mutants, the mutagenesis studies described hereafter were all based on hNaa60 (1-199).

### An amphipathic α-helix in the C-terminal region may contribute to Golgi localization of hNaa60

**Figure 2:**
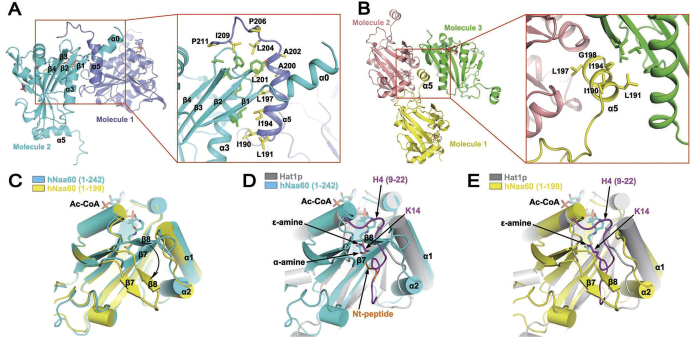
Amphipathicity of the α5 helix and alternative conformations of the β7-β8 hairpin. (**A**) The α5 helix of hNaa60(1-242) in one asymmetric unit (slate) interacts with another hNaa60 molecule in a neighboring asymmetric unit (cyan). A close view of the interaction is shown in red box. Side-chains of hydrophobic residues on α5 helix and the neighboring molecule participating in the interaction are shown as yellow and green sticks, respectively. (**B**) The α5 helix of hNaa60(1-199) in one asymmetric unit (yellow) interacts with another hNaa60 molecule in the neighboring asymmetric units (green). A close view of the interaction is shown in the red box. Side-chains of hydrophobic residues on α5 helix and the neighboring molecule (green) participating in the interaction are shown as yellow and green sticks, respectively. The third molecule (pink) does not directly interact with the α5 helix. (**C**) Superposition of hNaa60(1-199) (yellow) and hNaa60(1-242) (cyan) showing conformational change of the β7-β8 hairpin in these two structures. (**D,E**) Superposition of Hat1p/H4 (gray, drawn from PDB 4PSW) with hNaa60(1-242) (cyan, **D**) or hNaa60(1-199) (yellow, **E**). The histone H4 peptide (a KAT substrate) bound to Hat1p is shown in purple (**D,E**), while the peptide bound to hNaa50 (a NAT substrate, drawn from PDB 3TFY) is shown in orange (Nt-peptide) after superimposing hNaa50 (not shown in figure) on hNaa60 (**D**). The α-amine of the NAT substrate and ε-amine of the KAT substrate (along with the lysine side-chain) subject to acetylation are shown as sticks.

There is one hNaa60 molecule in the asymmetric unit in the hNaa60(1-242)/Ac-CoA structure. The C-terminal region extended from the GCN5-domain forms an amphipathic helix (α5) and interacts with a molecule in a neighbor asymmetric unit through hydrophobic interactions between α5-helix and a hydrophobic groove between the N-terminal β1 and β3 strands of the neighbor molecule (Fig. 2A). The C-terminal extension following α5-helix forms a β-turn that wraps around and interacts with the neighbor protein molecule through hydrophobic interactions, too. In the hNaa60(1-199)/CoA structure, a part of the α5-helix is deleted due to truncation of the C-terminal region (Fig. 1B). Interestingly, the remaining residues in α5-helix still form an amphipathic helix although the hydrophobic interaction with the N-terminal hydrophobic groove of a neighbor molecule is abolished and the helix is largely exposed in solvent due to different crystal packing (Fig. 2B).

A recent research showed that residues 182–216 are important for the localization of hNaa60 on Golgi^11^. According to our structure, the solvent-exposed amphipathic helix (α5) formed by residues 190-202 with an array of hydrophobic residues located on one side (Ile 190, Leu 191, Ile 194, Leu 197 and Leu 201) and hydrophilic residues on the other side (Fig. S2) might account for interaction between hNaa60 and Golgi membrane, as it is a typical structure accounting for membrane association through immersing into the lipid bi-layer with its hydrophobic side as was observed with KalSec14, Atg3, PB1-F2 *etc*^20^,^21^,^22^,^23^.

### The β7-β8 hairpin showed alternative conformations in the hNaa60 crystal structures

Superposition of hNaa60(1-242)/Ac-CoA, hNaa60(1-199)/CoA and hNaa50/CoA/peptide (PDB 3TFY) revealed considerable difference in the β7-β8 hairpin region despite the overall stability and similarity of the GNAT domain (Fig. 1D). In hNaa60(1-242), the β7-β8 hairpin is located in close proximity to the α1-α2 loop, creating a more compact substrate binding site than that in hNaa50, where this region adopts a more flexible loop conformation (β6-β7 loop). Upon removing the C-terminal region of hNaa60, we observed that hNaa60 (1-199) molecules pack in a different way involving the β7-β8 hairpin in the crystal, leading to about 50 degree rotation of the hairpin which moves away from the α1-α2 loop (Figs 1D and 2C).

This conformational change substantially altered the geometry of the substrate binding site, which could potentially change the way in which the substrate accesses the active site of the enzyme. In hNaa60(1-242), the β7-β8 hairpin covers the active site in a way similar to that observed in hNaa50, presumably leaving only one way for the substrate to access the active site, i.e. to enter from the opposite end into the same tunnel where Ac-CoA/CoA binds (Fig. 2D), which may accommodate access of a NAT substrate only. KAT activity of hNaa60 toward histone H4 has been noted in previous study^17^, and our enzyme kinetic data also indicated that hNaa60 can acetylate H3-H4 tetramer *in vitro* (Figure S3). Furthermore, we analyzed the acetylation status of histone H3-H4 tetramer using mass spectrometry and observed that multiple lysine residues in the protein showed significantly increased acetylation level and changed acetylation profile upon treatment with hNaa60(1-199) (Figure S4). We also conducted liquid chromatography-tandem mass spectrometry (LC/MS/MS) analysis on a synthetic peptide (NH_2_-MKGKEEKEGGAR-COOH) after treatment with hNaa60(1-199), and the data confirmed that both the N-terminal α-amine and lysine side-chain ε-amine were robustly acetylated after the treatment (Table S1). Despite these observations, the mechanism for this alternative activity remains unknown^17^. Recent structural investigation of other NATs proposed that the β6-β7 loop, corresponding to the β7-β8 hairpin in hNaa60, and the α1-α2 loop flanking the substrate-binding site of NATs, prevent the lysine side-chain of the KAT substrates from inserting into the active site^24^,^25^,^26^. Indeed, superposition of hNaa60(1-242) structure on that of Hat1p, a typical KAT, in complex with a histone H4 peptide^27^ revealed obvious overlapping/clashing of the H4 peptide (a KAT substrate) with the β7-β8 hairpin of hNaa60(1-242) (Fig. 2D). Interestingly, in the hNaa60(1-199) crystal structure, the displaced β7-β8 hairpin opened a second way for the substrate to access the active center that would readily accommodate the binding of the H4 peptide (Fig. 2E), thus implied a potential explanation for KAT activity of this enzyme from a structural biological view. However, since hNaa60(1-242) and hNaa60(1-199) were crystallized in different crystal forms, the observed conformational change of the β7-β8 hairpin may simply be an artifact related to the different crystal packing. Whether the KAT substrates bind to the β7-β8 hairpin displaced conformation of the enzyme needs to be verified by further structural and functional studies.

### Phe 34 facilitates proper positioning of the cofactor for acetyl-transfer

**Figure 3:**
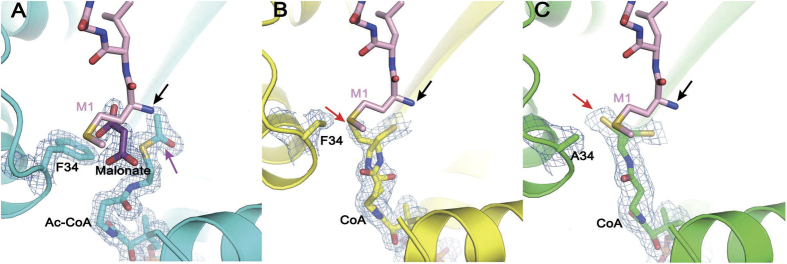
Electron density map of the active site. The 2Fo-Fc maps contoured at 1.0σ are shown for hNaa60(1-242)/Ac-CoA (**A**), hNaa60(1-199)/CoA (**B**) and hNaa60(1-199) F34A/CoA (**C**). The putative substrate peptide binding site is indicated by the peptide (shown as pink sticks) from the hNaa50/CoA/peptide complex structure after superimposing hNaa50 on the hNaa60 structures determined in this study. The black arrow indicates the α-amine of the first Met (M1) (all panels). The purple arrow indicates the acetyl moiety of Ac-CoA (**A**). The red arrow indicates the alternative conformation of the thiol moiety of the co-enzyme when Phe 34 side-chain is displaced (**B**) or mutated to Ala (**C**).

**Figure 4:**
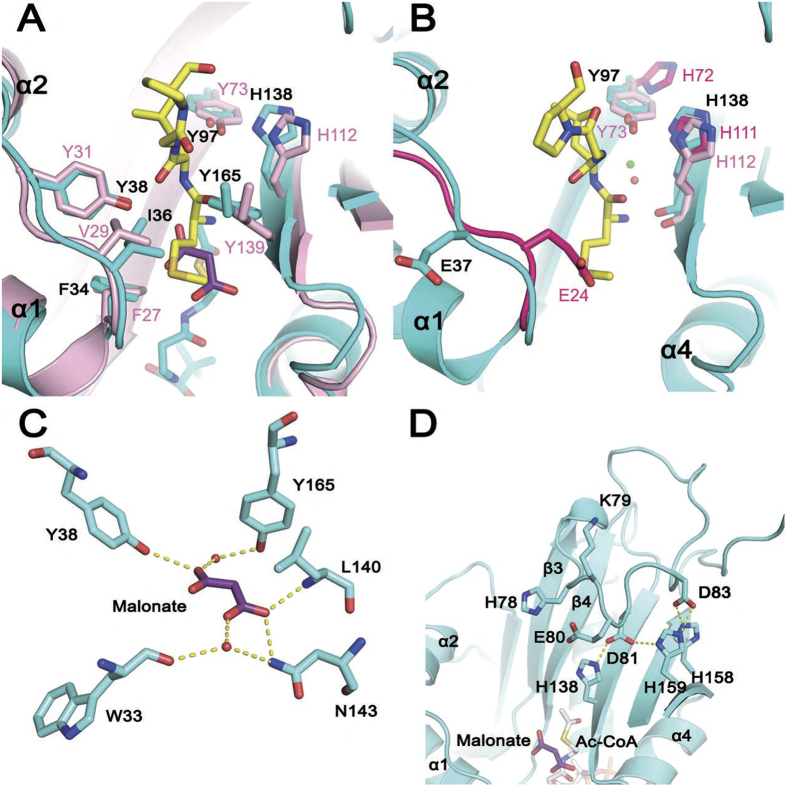
Structural basis for hNaa60 catalytic activity. (**A**) Superposition of hNaa60 active site (cyan) on that of hNaa50 (pink, PDB 3TFY). Side-chains of key catalytic and substrate-binding residues are highlighted as sticks. The malonate molecule in the hNaa60(1-242)/Ac-CoA structure and the peptide in the hNaa50/CoA/peptide structure are shown as purple and yellow sticks respectively. (**B**) A close view of the active site of hNaa60. Residues Glu 37, Tyr 97 and His 138 in hNaa60 (cyan) and corresponding residues (Tyr 73 and His 112) in hNaa50 (pink) as well as the side-chain of corresponding residues (Glu 24, His 72 and His 111) in complexed formed hNaa10p (warmpink) are highlighted as sticks. The water molecules participating in catalysis in the hNaa60 and hNaa50 structures are showed as green and red spheres, separately. (**C**) The interaction between the malonate molecule and surrounding residues observed in the hNaa60(1-242)/Ac-CoA structure. The yellow dotted lines indicate the hydrogen bonds. (**D**) A zoomed view of β3-β4 loop of hNaa60. Key residues discussed in the text (cyan), the malonate (purple) and Ac-CoA (gray) are shown as sticks. The yellow dotted lines indicate the salt bridges.

The electron density of Phe 34 side-chain is well defined in the hNaa60(1-242)/Ac-CoA structure, but becomes invisible in the hNaa60(1-199)/CoA structure, indicating displacement of the Phe 34 side-chain in the latter (Fig. 3A,B). A solvent-derived malonate molecule is found beside Phe 34 and the ethanethioate moiety of Ac-CoA in the high-resolution hNaa60(1-242)/Ac-CoA structure (Fig. 3A). Superposition of this structure on that of hNaa50p/CoA/peptide shows that the malonate molecule overlaps well on the N-terminal methionine of the substrate peptide and residue Phe 34 in hNaa60 overlaps well on Phe 27 in hNaa50 (Fig. 4A). Interestingly, in the structure of hNaa60(1-199)/CoA, the terminal thiol of CoA adopts alternative conformations. One is to approach the substrate amine (as indicated by the superimposed hNaa50/CoA/peptide structure), similar to the terminal ethanethioate of Ac-CoA in the structure of hNaa60(1-242)/Ac-CoA; the other is to approach the α1-α2 loop and away from the substrate amine (Fig. 3B). To rule out the possibility that the electron density we define as the alternative conformation of the thiol terminus is residual electron density of the displaced side-chain of Phe 34, we solved the crystal structure of hNaa60(1-199) F34A/CoA. The structure of this mutant is highly similar to hNaa60(1-199)/CoA and there is essentially the same electron density corresponding to the alternative conformation of the thiol (Fig. 3C).

**Figure 5:**
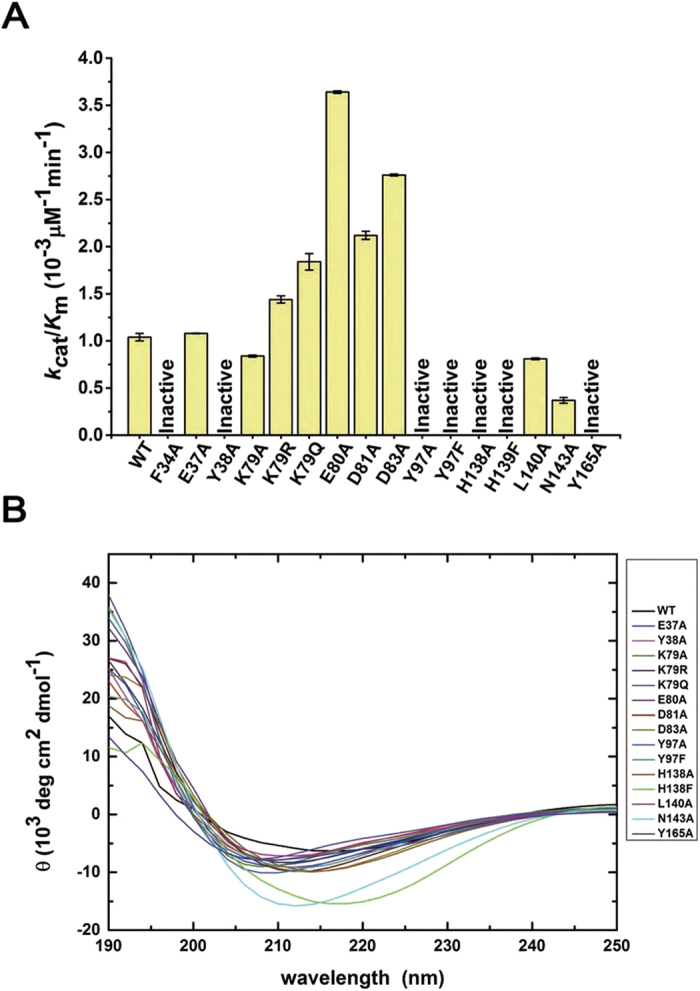
Catalytic activity of hNaa60 and mutant proteins. (**A**) Catalytic efficiency (shown as *k*_cat_/*K*_m_ values) of hNaa60 (1-199) WT and mutants. Error bars indicate the Standard Error (SE). (**B**) CD spectra of wild-type and mutant proteins from 250 nm to 190 nm. The sample concentration was 4.5 μM in 20 mM Tris, pH 8.0, 150 mM NaCl, 1% glycerol and 1 mM TCEP at room temperature.

Phe 27 in hNaa50p (equivalent to Phe 34 in hNaa60) has been implicated to facilitate the binding of N-terminal methionine of the substrate peptide through hydrophobic interaction^24^. However, in the hNaa60/Ac-CoA structure, a hydrophilic malonate molecule is found at the same location where the N-terminal methionine should bind as is indicated by the superposition (Fig. 3A), suggesting that Phe 34 may accommodate binding of hydrophilic substrate, too. Moreover, orientation of Phe 34 side-chain seems to be co-related to positioning of the terminus of the co-enzyme and important for placing it at a location in close proximity to the substrate amine. We hypothesize that if Phe 34 only works to facilitate the binding of the hydrophobic N-terminal Met residue, to mutate it from Phe to Ala would not abolish the catalytic activity of this enzyme, while if Phe 34 also plays an essential role to position the ethanethioate moiety of Ac-CoA, the mutation would be expected to abrogate the activity of the enzyme. Indeed, our enzyme kinetic data showed that hNaa60(1-199) F34A mutant showed no detectable activity (Fig. 5A). In order to rule out the possibility that the observed loss of activity may be related to bad folding of the mutant protein, we studied the circular dichroism (CD) spectrum of the protein (Fig. 5B) and determined its crystal structure (Fig. 3C). Both studies proved that the F34A mutant protein is well-folded. Many studies have addressed the crucial effect of α1-α2 loop on catalysis, showing that some residues located in this area are involved in the binding of substrates^24^,^25^,^26^,^28^. We propose that Phe 34 may play a dual role both in interacting with the peptide substrate (recognition) and in positioning of the ethanethioate moiety of Ac-CoA to the right location to facilitate acetyl-transfer.

### Structural basis for hNaa60 substrate binding

Several studies have demonstrated that the substrate specificities of hNaa60 and hNaa50 are highly overlapped^9^,^12^,^18^. The structure of hNaa50p/CoA/peptide provides detailed information about the position of substrate N-terminal residues in the active site of hNaa50^24^. Comparing the active site of hNaa60(1-242)/Ac-CoA with hNaa50p/CoA/peptide revealed that key catalytic and substrate binding residues are highly conserved in both proteins (Fig. 4A). With respect to catalysis, hNaa50p has been shown to employ residues Tyr 73 and His 112 to abstract proton from the α-amino group from the substrate’s first residue through a well-ordered water^24^. A well-ordered water was also found between Tyr 97 and His 138 in hNaa60 (1-199)/CoA and hNaa60 (1-242)/Ac-CoA (Fig. 4B). To determine the function of Tyr 97 and His 138 in hNaa60 catalysis, we mutated these residues to alanine and phenylalanine, respectively, and confirmed that all these mutants used in our kinetic assays are well-folded by CD spectra (Fig. 5B). Purity of all proteins were also analyzed by SDS-PAGE (Figure S5). As show in Fig. 5A, the mutants Y97A, Y97F, H138A and H138F abolished the activity of hNaa60. In contrast, to mutate the nearby solvent exposed residue Glu 37 to Ala (E37A) has little impact on the activity of hNaa60 (Figs 4B and 5A). In conclusion, the structural and functional studies indicate that hNaa60 applies the same two base mechanism through Tyr 97, His 138 and a well-ordered water as was described for hNaa50^24^.

The malonate molecule observed in the hNaa60(1-242)/Ac-CoA crystal structure may be indicative of the substrate binding position of hNaa60 since it is located in the active site and overlaps the N-terminal Met of the substrate peptide in the superposition with the hNaa50p/CoA/peptide structure (Fig. 4A). Residues Tyr 38, Asn 143 and Tyr 165 are located around the malonate and interact with it through direct hydrogen bonds or water bridge (Fig. 4C). Although malonate is negatively charged, which is different from that of lysine ε-amine or peptide N-terminal amine, similar hydrophilic interactions may take place when substrate amine presents in the same position, since Tyr 38, Asn 143 and Tyr 165 are not positively or negatively charged. In agreement with this hypothesis, it was found that the Y38A, N143A and Y165A mutants all showed remarkably reduced activities as compared to WT, implying that these residues may be critical for substrate binding (Figs 4C and 5A).

### The β3-β4 loop participates in the regulation of hNaa60-activity

Residues between β3 and β4 of hNaa60 form a unique 20-residue long loop (residues 73–92) that is a short turn in many other NAT members (Fig. 1D). Previous study indicated that auto-acetylation of hNaa60K79 could influence the activity of hNaa60^17^; however, we were not able to determine if Lys 79 is acetylated in our crystal structures due to poor quality of the electron density of Lys 79 side-chain. We therefore used mass spectrometry to analyze if Lys 79 was acetylated in our bacterially purified proteins, and observed no modification on this residue (Figure S6). To assess the impact of hNaa60K79 auto-acetylation, we studied the kinetics of K79R and K79Q mutants which mimic the un-acetylated and acetylated form of Lys 79, respectively. Interestingly, both K79R and K79Q mutants led to an increase in the catalytic activity of hNaa60, while K79A mutant led to modest decrease of the activity (Fig. 5A). These data indicate that the acetylation of Lys 79 is not required for optimal catalytic activity of hNaa60 *in vitro*.

It is noted that the β3-β4 loop of hNaa60 acts like a door leaf to partly cover the substrate-binding pathway. We hence hypothesize that the β3-β4 loop may interfere with the access of the peptide substrates and that the solvent-exposing Lys 79 may play a potential role to remove the door leaf when it hovers in solvent (Fig. 4D). Acidic residues Glu 80, Asp 81 and Asp 83 interact with His 138, His 159 and His 158 to maintain the conformation of the β3-β4 loop, thus contribute to control the substrate binding (Fig. 4D). To verify this hypothesis, we mutated Glu 80, Asp 81 and Asp 83 to Ala respectively. In line with our hypothesis, E80A, D81A and D83A mutants exhibit at least 2-fold increase in hNaa60-activity (Fig. 5A). Interestingly, the structure of an ancestral NAT from *S. solfataricus* also exhibits a 10-residue long extension between β3 and β4, and the structure and biochemical studies showed that the extension of SsNat has the ability to stabilize structure of the active site and potentiate SsNat-activity^28^.

## Discussion

Nt-acetylation, which is carried out by the NAT family acetyltransferases, is an ancient and essential modification of proteins. Although many NATs are highly conserved from lower to higher eukaryotes and the substrate bias of them appears to be partially overlapped, there is a significant increase in the overall level of N-terminal acetylation from lower to higher eukaryotes^18^. In this study we provide structural insights into Naa60 found only in multicellular eukaryotes.

The N-terminus of hNaa60 harbors three hydrophobic residues (VVP) that makes it very difficult to express and purify the protein. This problem was solved by replacing residues 4–6 from VVP to EER that are found in Naa60 from *Xenopus Laevis*. Since Naa60 from human and from *Xenopus Laevis* are highly homologous (Fig. 1A), we speculate that these two proteins should have the same biological function. Therefore it is deduced that the VVP to EER replacement on the N-terminus of hNaa60 may not interfere with its function. However, in the hNaa60(1-242) structure the N-terminus adopts an α-helical structure which will probably be kinked if residue 6 is proline (Fig. 1C), and in the hNaa60(1-199) structure the N-terminus adopts a different semi-helical structure (Fig. 1B) likely due to different crystal packing. Hence it is not clear if the N-terminal end of wild-type hNaa60 is an α-helix, and what roles the hydrophobic residues 4–6 play in structure and function of wild-type hNaa60. In addition to the three-residue mutation (VVP to EER), we also tried many other hNaa60 constructs, but only the full-length protein and the truncated variant 1-199 behaved well. The finding that the catalytic activity of hNaa60(1-242) is much lower than that of hNaa60(1-199) is intriguing. We speculate that low activity of the full-length hNaa60 might be related to lack of Golgi localization of the enzyme in our *in vitro* studies or there remains some undiscovered auto-inhibitory regulation in the full-length protein.

The hNaa60 protein was proven to be localized on Golgi apparatus^11^,^17^. Aksnes and colleagues predicted putative transmembrane domains and two putative sites of S-palmitoylation, by bioinformatics means, to account for Golgi localization of the protein. They then mutated all five cysteine residues of hNaa60’s to serine, including the two putative S-palmitoylation sites. However, these mutations did not abolish Naa60 membrane localization, indicating that S-palmitoylation is unlikely to (solely) account for targeting hNaa60 on Golgi. Furthermore, adding residues 217–242 of hNaa60 (containing residues 217–236, one of the putative transmembrane domains) to the C terminus of eGFP were not sufficient to localize the protein on Golgi apparatus, while eGFP-hNaa60_182-242_ was sufficient to, suggesting that residues 182–216 are important for Golgi localization of hNaa60. We found that residues 190–202 formed an amphipathic helix with an array of hydrophobic residues located on one side. This observation is reminiscent of the protein/membrane interaction through amphipathic helices in the cases of KalSec14, Atg3, PB1-F2 *etc*^20^,^21^,^22^,^23^. In this model an amphipathic helix can immerse its hydrophobic side into the lipid bilayer through hydrophobic interactions. Therefore we propose that the amphipathic helix α5 may contribute to Golgi localization of hNaa60. This model, though may need further studies, is supported by the Aksnes studies.

Previous studies indicated that members of NAT family are bi-functional NAT and KAT enzymes. However, known structures of NATs do not well support this hypothesis, since the β6-β7 hairpin/loop of most of NATs is involved in the formation of a tunnel-like substrate-binding site with the α1-α2 loop, which would be good for the NAT but not KAT activity of the enzyme^9^,^26^. Kinetic studies have been conducted to compare the NAT and KAT activity of hNaa50 *in vitro*, and indicate that the NAT activity of Naa50 is much higher than KAT activity^16^. However, the substrate used in this study for assessing KAT activity was a small peptide which could not really mimic the 3D structure of a folded protein substrate *in vivo*. Our mass spectrometry data indicated that there were robust acetylation of histone H3-H4 tetramer lysines and both N-terminal acetylation and lysine acetylation of the peptide used in the activity assay, thus confirmed the KAT activity of this enzyme *in vitro*. Conformational change of the β7-β8 hairpin (corresponding to the β6-β7 loop of other NATs) is noted in our structures (Figs 1D and 2C), which might provide an explanation to the NAT/KAT dual-activity in a structural biological view, but we were unable to rule out the possibility that the observed conformational change of this hairpin might be an artifact related to crystal packing or truncation of the C-terminal end of the protein. Further studies are therefore needed to reveal the mechanism for the KAT activity of this enzyme.

The relationship between enzyme, co-enzyme and substrates has been documented for several years. In early years, researchers found adjustment of GCN5 histone acetyltransferase structure when it binds CoA molecule^29^. The complexed form of NatA is more suitable for catalytic activation, since the α1-α2 loop undergoes a conformation change to participate in the formation of substrate-binding site when the auxiliary subunit Naa15 interacts with Naa10 (the catalytic subunit of NatA)^25^. In the structure of hNaa50/CoA/peptide, Phe 27 in the α1-α2 loop appears to make hydrophobic interaction with the N-terminal Met of substrate peptide^24^. However, the hNaa60(1-242)/Ac-CoA crystal structure indicated that its counterpart in hNaa60, Phe 34, could also accommodate the binding of a hydrophilic malonate that occupied the substrate binding site although it maintained the same conformation as that observed in hNaa50. Interestingly, the terminal thiol of CoA adopted alternative conformations in the structure of hNaa60(1-199)/CoA. One was to approach the substrate amine; the other was to approach the α1-α2 loop and away from the substrate amine. Same alternative conformations of CoA were observed in the hNaa60(1-199)(F34A) crystal structure, and our kinetic data showed that the F34A mutation abolished the activity of the enzyme. Taken together, our data indicated that Phe 34 in hNaa60 may play a role in placing co-enzyme at the right location to facilitate the acetyl-transfer. However, these data did not rule out that possibility that Phe 34 may coordinate the binding of the N-terminal Met through hydrophobic interaction as was proposed by previous studies^24^.

Furthermore, we showed that hNaa60 adopts the classical two base mechanism to catalyze acetyl-transfer. Although sequence identity between hNaa60 and hNaa50 is low, key residues in the active site of both enzymes are highly conserved. This can reasonably explain the high overlapping substrates specificities between hNaa60 and hNaa50. Another structural feature of hNaa60 that distinguishes it from other NATs is the β3-β4 long loop which appears to inhibit the catalytic activity of hNaa60. However, this loop also seems to stabilize the whole hNaa60 structure, because deletion mutations of this region led to protein precipitation and aggregation (Figure S7). A previous study suggested that the auto-acetylation of Lys 79 was important for hNaa60-activity^17^, whereas the point mutation K79R did not decrease the activity of hNaa60 in our study. Meanwhile, no electron density of acetyl group was found on Lys 79 in our structures and mass spectrometry analysis. Hence, it appears that the auto-acetylation of hNaa60 is not an essential modification for its activity for the protein we used here. As for the reason why K79R in Yang’s previous studies reduced the activity of the enzyme^17^, but in our studies it didn’t, we suspect that the stability of this mutant may play some role. K79R is less stable than the wild-type enzyme as was judged by its poorer gel-filtration behavior and tendency to precipitate. In our studies we have paid special attention and carefully handled this protein to ensure that we did get enough of the protein in good condition for kinetic assays. The intracellular environment is more complicated than our *in vitro* assay and the substrate specificity of hNaa60 most focuses on transmembrane proteins. The interaction between hNaa60 and its substrates may involve the protein-membrane interaction which would further increase the complexity. It is not clear if the structure of hNaa60 is different *in vivo* or if other potential partner proteins may help to regulate its activity. Nevertheless, our study may be an inspiration for further studies on the functions and regulation of this youngest member of the NAT family.

## Methods

### Cloning, expression and purification of *Homo sapiens* Naa60 (hNaa60)

The cDNA encoding hNaa60 residues 1–242 (full-length) or residues 1–199 were amplified by PCR and inserted into the pET23a vector, which had been modified to provide an N-terminal 6xHis-tag followed by a tobacco etch virus (TEV) protease cleavage site. The VVP to EER (residues 4–6) mutation and other mutations for functional studies were introduced using the quick change method. The protein was expressed in *Escherichia coli* BL21 (DE3) or *Escherichia coli* BL21 (DE3) pLysS at 16 °C for 15 h in the presence of 0.1 mM IPTG. Cells were harvested at 4 °C by centrifugation (4,000 g for 10 min) and resuspended in buffer A containing 20 mM Tris, pH 8.0, 500 mM NaCl, 50 mM imidazole, 10% glycerol, 1 mM protease inhibitor PMSF (Phenylmethylsulfonyl fluoride) and 1 mM Tris (2-carboxyethyl)phosphine (TCEP) hydrochloride. Cells were lysed by sonication and the lysate was cleared by centrifugation (18,000 g at 4 °C for 20 min). Then the supernatant was loaded onto a 5-mL Chelating Sepharose column (GE Healthcare) charged with Ni^2+^ and washed with buffer B (20 mM Tris, pH 8.0, 500 mM NaCl, 50 mM imidazole, 1% glycerol and 1 mM TCEP). The protein was eluted with buffer C (20 mM Tris, pH 8.0, 500 mM NaCl, 300 mM imidazole, 1% glycerol and 1 mM TCEP). The eluent was digested by His-tagged TEV protease and concentrated by ultrafiltration at the same time. After 3 hours, the concentrated eluent was diluted 10 times with buffer D (20 mM Tris, pH 8.0, 500 mM NaCl, 1% glycerol and 1 mM TCEP) and the diluent was passed through the nickel column once again to remove the His-tagged TEV protease and the un-cleaved His-hNaa60 protein. The flow-through was concentrated to 500 μl and loaded onto a Superose 6 or Superdex 200 10/300 gel-filtration column (GE Healthcare) equilibrated with buffer E (20 mM Tris, pH 8.0, 150 mM NaCl, 1% glycerol and 1 mM TCEP). Fractions containing the protein were collected and concentrated to a final concentration of 10 mg/ml for crystallization or acetyltransferases assays.

### Circular Dichroism (CD) Spectroscopy

CD spectra of the proteins were obtained using a Jasco J-810 circular dichroism spectropolarimeter scanning from 190 to 250 nm with a 1 mm quartz cuvette. The wild-type and mutant proteins were examined at 4.5 μM concentration in 20 mM Tris, pH 8.0, 150 mM NaCl, 1% glycerol and 1 mM TCEP at room temperature. All samples were centrifuged at 10,000 g for 5 min before analysis.

### Crystallization, data collection and structure determination

The purified hNaa60(1-242), hNaa60(1-199) or F34A(1-199) protein was mixed with acetyl coenzyme A (Ac-CoA) or coenzyme A (CoA) (Sigma), respectively, at a 1:5 molar ratio before crystallization. All crystals were made by the hanging-drop vapor diffusion method. The crystallization reservoir solution for hNaa60(1-242) was 10 mM Tris pH 8.0, 75 mM NaCl, 0.5% glycerol, 3% v/v Tacsimate pH 4.0 (Hampton Research) and 7.5% w/v polyethylene glycol 3350 (PEG 3350), and for hNaa60(1-199) was 0.2 M L-Proline, 0.1 M HEPES pH 7.5, 10% w/v PEG 3350. Crystals of F34A mutation were obtained in 0.2 M Lithium Sulfate monohydrate, 0.1 M Tris pH 8.5, 20% w/v PEG 3350. The crystals were flash-frozen in liquid nitrogen in a cryo-protectant made of the reservoir solution supplemented with 25% glycerol.

The diffraction data were collected at the Shanghai SSRF BL18U1 beamline or at the Argonne National Laboratory APS ID19 beamline at 100 K. The data were processed with HKL3000^30^. The hNaa60(1-199) structure was determined by molecular replacement with Phaser^31^ using a previously reported GNAT family acetyltransferase structure (PDB 2AE6) as the search model. The hNaa60(1-242) structure was solved by molecular replacement using hNaa60(1-199) structure as the search model. To improve the model quality, the programs ARP/wARP^32^ in CCP4 or simulated-annealing in CNS^33^ were used. Iterative cycles of manual refitting and crystallographic refinement were performed using COOT^34^ and Phenix^35^. Ac-CoA/CoA and malonate were modeled into the closely fitting positive Fo-Fc electron density and then included in following refinement cycles. Topology and parameter files for Ac-CoA/CoA and malonate were generated using PRODRG^36^. All figures for the molecular models were prepared using the PyMOL program. Statistics of diffraction data processing and structure refinement are shown in Table 1.

### Acetyltransferase assay

Acetyltransferase assay of hNaa60 was conducted as described previously^37^. Briefly, a reaction cocktail containing 100 mM Tris-HCl buffer, pH 8.5, 0.07% alkylated BSA, 0.01% NP-40, 1 mM EDTA, 150 μM Ac-CoA (Sigma) was prepared and varied concentrations of the substrate peptide (0–400 μM) (NH_2_-MKGKEEKEGGAR-COOH) was added in a 1.5-mL microfuge tube, and then the respective enzyme was added to initiate the reaction with a final assay volume of 100 μL. The reaction was carried out for 20 minutes at 37 °C. Aliquots (40 μL) of the reaction were then removed and quenched with 40 μL of ice-cold isopropanol in individual wells of a 96-well black microplate (Corning), and then mixed with 80 μl of 25 μM 7-diethylamino-3-(49 maleimidylphenyl)-4-methylcoumarin (CPM) (Sigma) in 100 mM Tris-HCl (pH 8.5) and 1% Triton X-100 and allowed to react in darkness for 10 minutes prior to reading. The fluorescence signal was monitored using a Varioskan Flash plate reader (Thermo Scientific) at Ex_max_ = 385 nm and Em_max_ = 465 nm. Substrate inhibition appeared at high concentrations of substrate peptide prevented our kinetics assays from reaching saturation of the enzyme. Therefore, we determined the value of *k*_cat_/*K*_m_ by fitting our data to the equation: v = (*k*_cat_/*K*_m_)[E_T_][S] when the substrate concentration was far less than *K*_m_. The assays were done in triplicate. The slope of the line indicates the *k*_cat_/*K*_m_ value of the enzyme (Figure S1).

## Additional Information

**How to cite this article**: Chen, J.-Y. *et al*. Structure and function of human Naa60 (NatF), a Golgi-localized bi-functional acetyltransferase. *Sci. Rep.* **6**, 31425; doi: 10.1038/srep31425 (2016).

## Supplementary Material

Supplementary Information

## Figures and Tables

**Table 1:** Data collection and refinement statistics.

Structure and PDB ID	hNaa60(1-242)/Ac-CoA 5HGZ	hNaa60(1-199)/CoA 5HH0	hNaa60(1-199)F34A/CoA 5HH1
Data collection*			
Space group	*P2*_*1*_*2*_*1*_*2*_*1*_	*P2*_*1*_*2*_*1*_*2*	*P2*_*1*_*2*_*1*_*2*
Cell dimensions			
*a, b, c* (Å)	53.3, 57.4, 68.8	67.8, 73.8, 43.2	66.7, 74.0, 43.5
α,β,γ (°)	90.0, 90.0, 90.0	90.0, 90.0, 90.0	90.0, 90.0, 90.0
Resolution (Å)	50–1.38 (1.42–1.38)	50–1.60 (1.66–1.60)	50–1.80 (1.86–1.80)
*R*_p.i.m._(%)**	3.0 (34.4)	2.1 (32.5)	2.6 (47.8)
*I*/*σ*	21.5 (2.0)	31.8 (2.0)	28.0 (2.4)
Completeness (%)	99.8 (99.1)	99.6 (98.5)	99.9 (99.7)
Redundancy	6.9 (5.0)	6.9 (6.2)	6.3 (5.9)
Refinement			
Resolution (Å)	25.81–1.38	33.55–1.60	43.52–1.80
No. reflections	43660	28588	20490
* R*_work_/*R*_free_	0.182/0.192	0.181/0.184	0.189/0.209
No. atoms			
Protein	1717	1576	1566
Ligand/ion	116	96	96
Water	289	258	168
*B*-factors			
Protein	23.8	32.0	37.4
Ligand/ion	22.2	34.6	43.7
Water	35.1	46.4	49.1
R.m.s. deviations			
Bond lengths (Å)	0.018	0.017	0.015
Bond angles (°)	1.529	1.651	1.581
Ramachandran Plot			
Favoured region	98.98%	98.93%	98.96%
Allowed region	1.02%	1.07%	1.04%
Outliers	0.00%	0.00%	0.00%

^*^Values in parentheses are for highest-resolution shell. One crystal was used for each data set.

^**^R_p.i.m._, a redundancy-independent *R* factor was used to evaluate the diffraction data quality as was proposed by Evans^38^.
