# Supplementary material for: Structure and function of human Naa60 (NatF), a Golgi-localized bi-functional acetyltransferase
Source: Sci Rep. 2016 Aug 23;6:31425. doi: 10.1038/srep31425 (PMC4993997; doi:10.1038/srep31425)
Supplement: Supplementary Information [file srep31425-s1.pdf]

Supplemental Information

Structure and function of human Naa60 (NatF), a Golgi-localized bi-functional acetyltransferase

Ji-Yun Chen, Liang Liu, Chun-Ling Cao, Mei-Jun Li, Kemin Tan, Xiaohan Yang, and Cai-Hong Yun

Supplemental Figures

Figure S1. Catalytic efficiency of hNaa60(1-199) wild-type and mutant proteins. The assays were done in triplicate. The error bars indicate standard deviation (SD) of every dot. The slope of the line indicates the k_{cat}/K_m value of the enzyme.

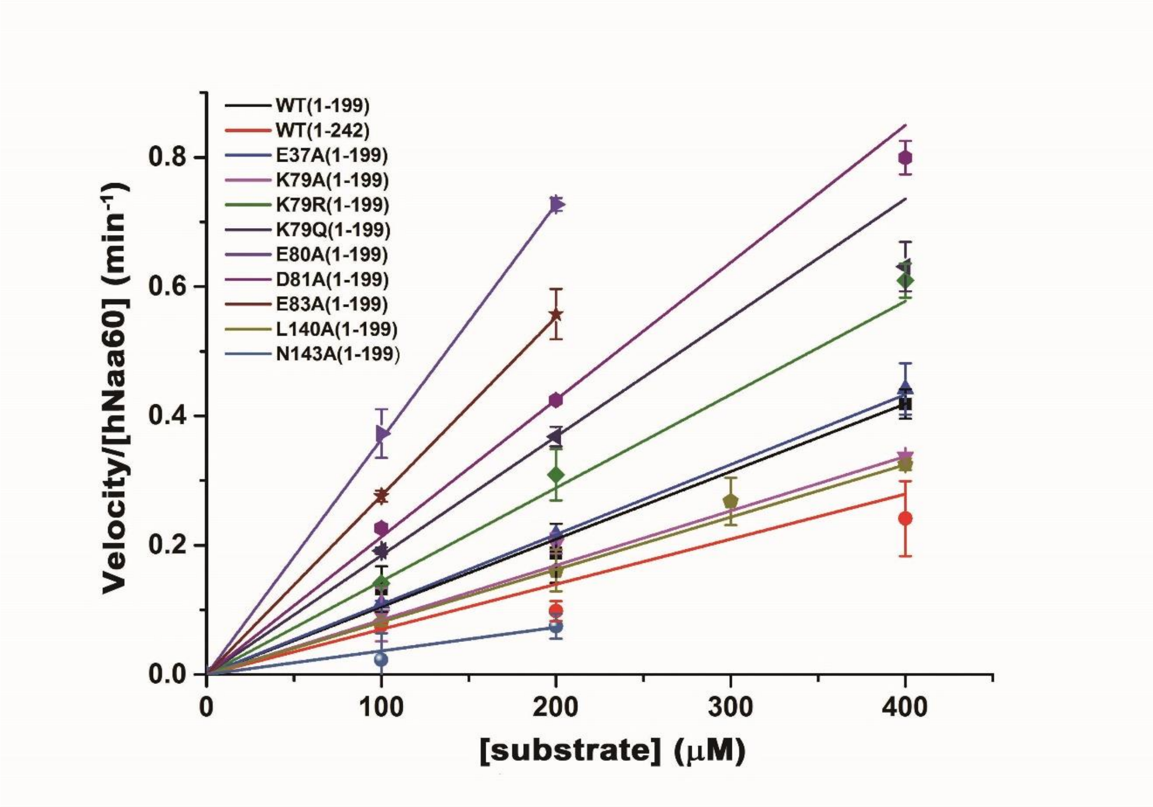

Figure S2. **The helical wheel projection diagram of α5 helix of hNaa60.** Different colors are used to indicate residue property: yellow, typical hydrophobic residues; blue, basic residues; red, acidic residues; purple, uncharged hydrophilic residues; light-red, tyrosine and light-yellow, glycine.

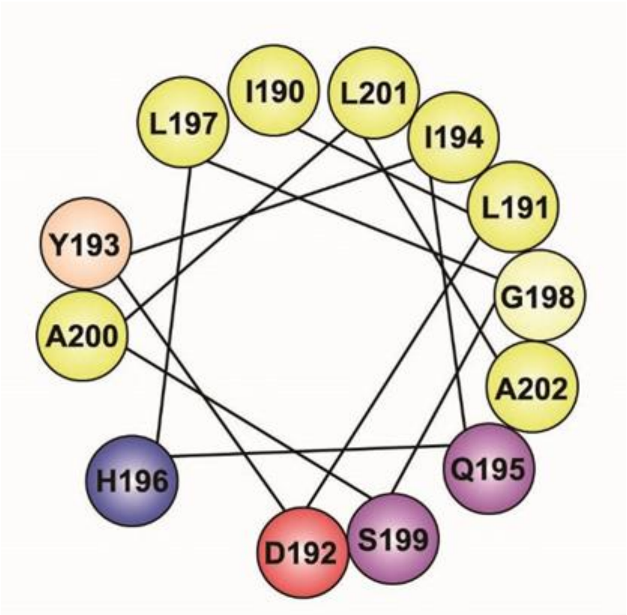

Figure S3. Naa60(1-199) can acetylate H3-H4 tetramer in vitro. Recombinant human Naa60 and human H3-H4 tetramer were used in these experiments. Recombinant human H3-H4 tetramer were purified as described[1] with minor modification. The hNaa60(1-199) protein (1 μM) was mixed with Ac-CoA (240 μM) and H3-H4 tetramer (14 μM) and incubated in the reaction buffer (100 mM HEPES pH7.5, 100 mM NaCl) in a total volume of 50 μl for 1h at 37 °C. The control group did not contain the enzyme. The concentration measurement of CoA was the same as the NAT-activity assay of hNaa60. Experiments were carried out in triplicate and error bars indicate standard deviation (SD).

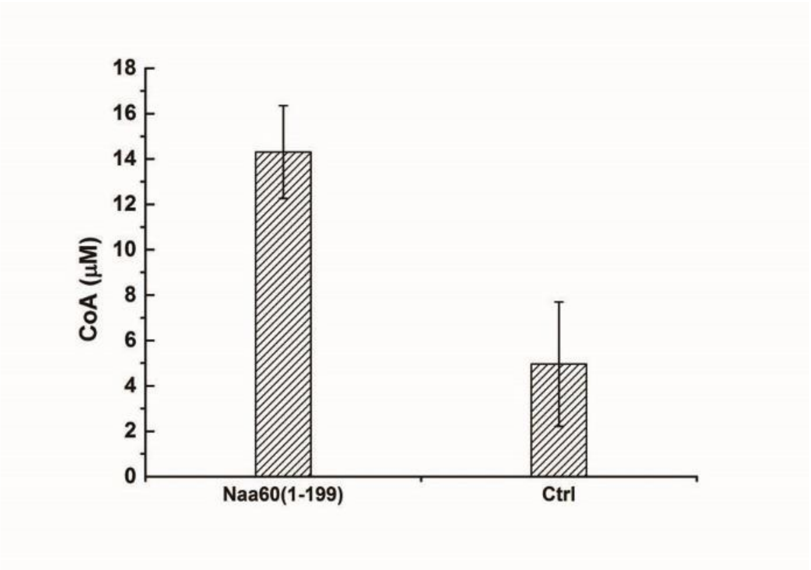

Sample treatment	CoA production (μM)
H3-H4 plus Ac-CoA and Naa60(1-199)	14.31±2.05
H3-H4 plus Ac-CoA	4.96±2.74

Figure S4: Mass spectrometry analysis of acetylation status of H3-H4 tetramer with and without treatment with hNaa60(1-199). Recombinant human Naa60 and human H3-H4 tetramer were used in the study. The hNaa60(1-199) protein (1 μM) was mixed with Ac-CoA (240 μM) and H3-H4 tetramer (14 μM) and incubated in the reaction buffer (100 mM HEPES pH7.5, 100 mM NaCl) in a total volume of 50 μl for 1 hour at 37 ℃. The control group did not contain the enzyme. After terminating the reaction, the same amount of treated and untreated H3-H4 samples were applied to mass spectrometry analysis. All acetylated peptides detected by mass spectrometry were shown. Although mass spectrometry cannot accurately quantify different peptides due to different ionization efficiency *etc.*, the remarkably higher overall acetylation level and the changed acetylation profile of the hNaa60-treated sample compared to the un-treated sample indicated that hNaa60(1-199) does have KAT activity toward the H3-H4 tetramer *in vitro*.

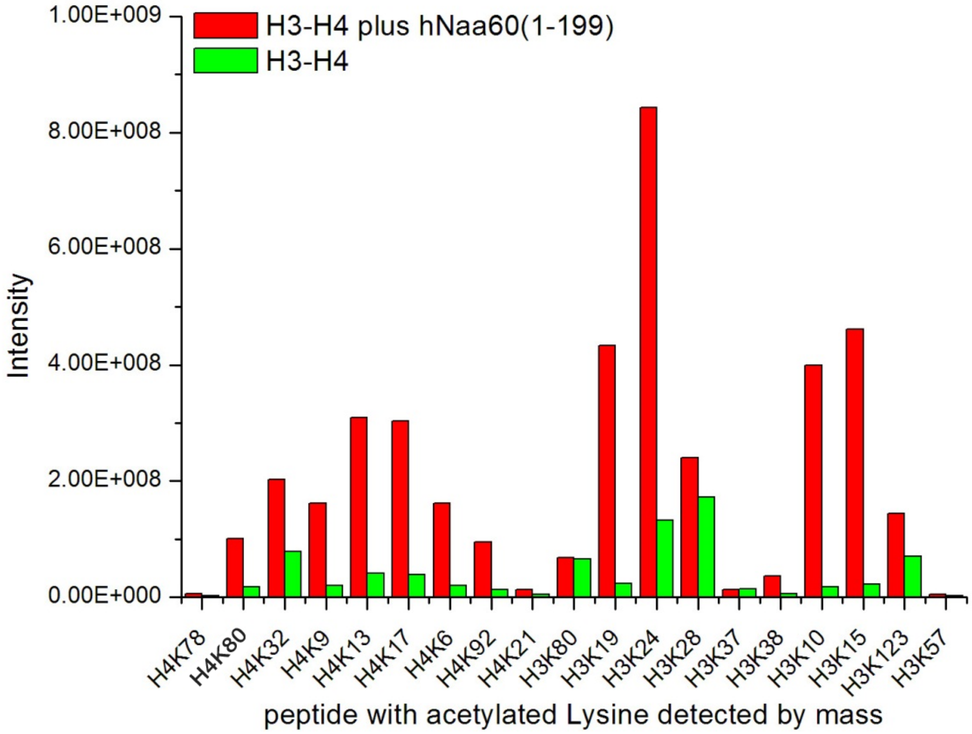

Figure S5: Purity of the proteins analyzed by SDS-PAGE.

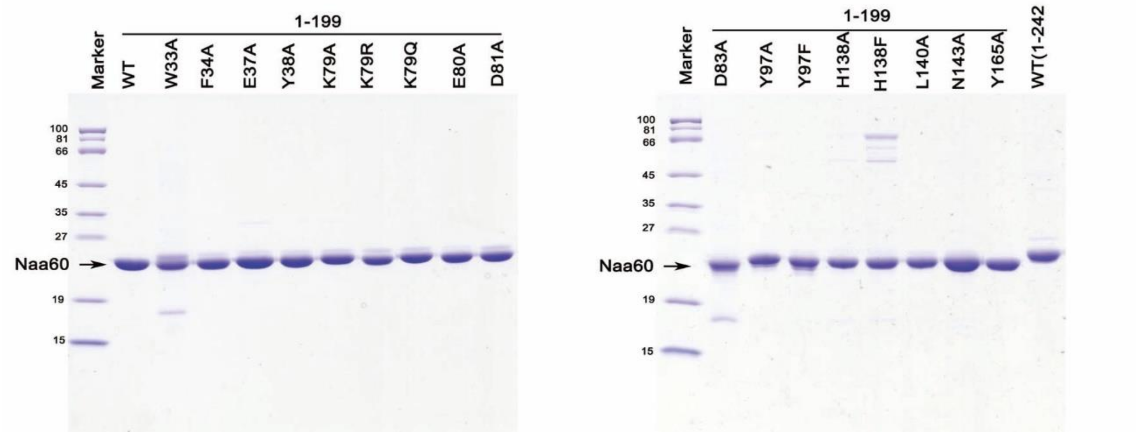

Figure S6. Mass spectrometry analysis of lysine residues of bacterially expressed hNaa60 (1-199).

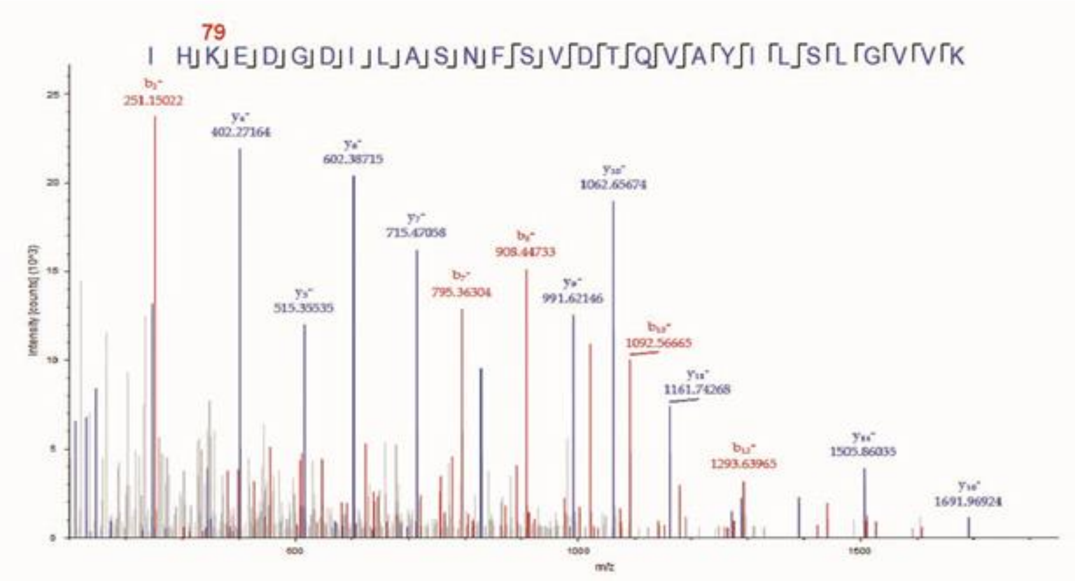

Figure S7. Deletion mutations of β3-β4 loop led to protein precipitation and aggregation. The β3-β4 loop (74-93 aa) were replaced by corresponding residues (SQNQ) of hNaa50p. The purification of hNaa60(1-199&△74-93 SQNQ) was analyzed by Size-exclusion chromatography (A) and SDS-PAGE assay (B).

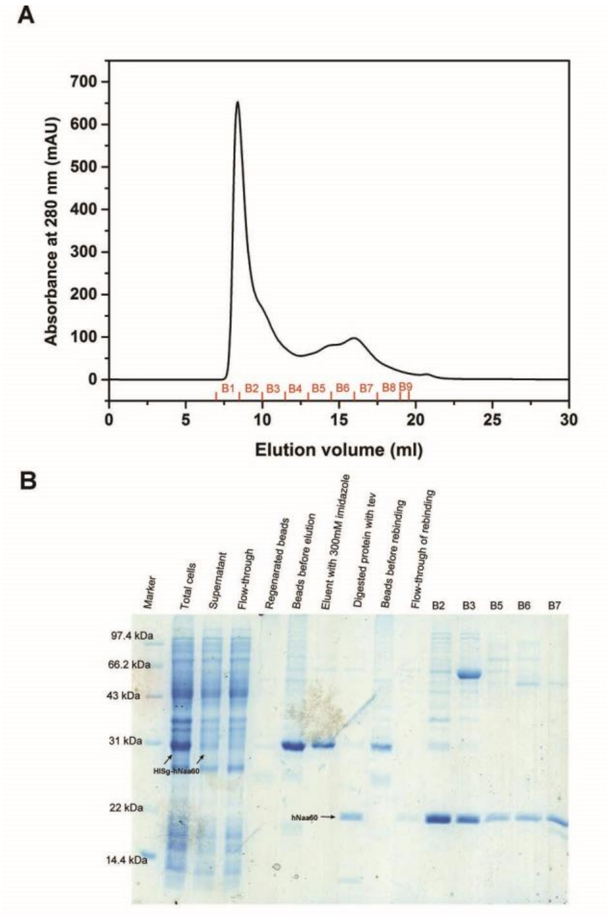

Supplemental Tables

Table S1. Acetylation status of peptide NH_2-MKGKEEKEGGAR-COOH after treatment with hNaa60(1-199) analyzed by LC/MS/MS.

peptide	intensity	modification	activity
EKEGGAR	1.268E6	(none)	
GKEEKEGGAR	1.038E7	N-Term(Acetyl)	NAT
GKEEKEGGAR	7.004E8	(none)	
KEEKEGGAR	7.396E7	(none)	
KGKEEKEGGAR	9.079E6	N-Term(Acetyl)	NAT/KAT
KGKEEKEGGAR	3.419E5	K4(Acetyl); K7(Acetyl)	KAT
KGKEEKEGGAR	1.228E9	(none)	
MKGKEEKEGG	1.492E7	(none)	
MKGKEEKEGGA	9.300E6	N-Term(Acetyl); K2(Acetyl)	NAT+KAT
MKGKEEKEGGA	1.137E8	N-Term(Acetyl)	NAT
MKGKEEKEGGA	2.660E7	(none)	
MKGKEEKEGGAR	3.944E7	M1(Oxidation); K2(Acetyl); K4(Acetyl)	KAT
MKGKEEKEGGAR	2.669E6	M1(Oxidation); K2(Acetyl)	KAT
MKGKEEKEGGAR	1.949E9	N-Term(Acetyl); K2(Acetyl)	NAT+KAT
MKGKEEKEGGAR	2.923E7	N-Term(Acetyl); K4(Acetyl); K7(Acetyl)	NAT+KAT
MKGKEEKEGGAR	3.060E9	M1(Oxidation)	
MKGKEEKEGGAR	1.988E10	K2(Acetyl)	KAT
MKGKEEKEGGAR	1.773E10	(none)	

Reference:

1 Tanaka, Y. *et al.* Expression and purification of recombinant human histones. *Methods* **33**, 3-11, doi:10.1016/j.ymeth.2003.10.024 (2004).
